# Indication, Location of the Lesion, Diagnostic Yield, and Therapeutic Yield of Double-Balloon Enteroscopy: Seventeen Years of Experience

**DOI:** 10.3390/diagnostics12092224

**Published:** 2022-09-14

**Authors:** Sang Pyo Lee, Hyun Joo Jang, Sea Hyub Kae, Jae Gon Lee, Ji Hye Kwon

**Affiliations:** Division of Gastroenterology, Department of Internal Medicine, Hallym University Dongtan Sacred Heart Hospital, Hallym University College of Medicine, 7 Keunjaebong-gil, Hwaseong 18450, Korea

**Keywords:** double-balloon enteroscopy, balloon enteroscopy, small intestine

## Abstract

Double-balloon enteroscopy (DBE) has become one of the standard methods in the diagnosis and treatment of small bowel (SB) disease. However, previous studies for DBE have limitations due to heterogeneity of indications and operators. The aim was to investigate the indication, location of the lesion, diagnostic yield, and therapeutic yield of DBE based on long-term data from a single operator. A retrospective study was performed by reviewing medical records of subjects who had received DBE at our unit in the past 17 years. Overall diagnostic yield was 78.7% (210/267). The diagnostic yield for obscure gastrointestinal bleeding (OGIB) was 68.3% (84/123). The diagnostic yield for OGIB was significantly lower (*p* < 0.001) than that for other indications. Therapeutic yield was 24.7% (66/267). Complications occurred in 7 (2.6%). Crohn’s disease, intestinal tuberculosis, nonsteroidal anti-inflammatory drug enteropathy, and diverticular lesions were mainly found in the ileum. Vascular lesions, non-specific inflammation, and neoplastic lesions were found more frequently in the jejunum. DBE is an excellent and safe endoscopic method for the diagnosis and treatment of SB lesions. DBE has a lower diagnostic rate for OGIB than for other indications. The location where a lesion is commonly found depends on the type of the lesion.

## 1. Introduction

Diagnosis and treatment for small intestinal lesions cannot be performed with conventional endoscopy. Thus, various diagnostic and therapeutic methods have been developed. Device-assisted enteroscopy, which is a generic term for endoluminal examination of the small bowel with an endoscopic technique, can be divided into two groups: balloon enteroscopy and spiral enteroscopy [1,2]. The former is based on the push-and-pull principle and the latter is based on the principle of rotation [3]. Balloon enteroscopy can be further subdivided into balloon-guided enteroscopy and balloon-assisted enteroscopy (BAE), which can be performed with either a single-balloon enteroscopy (SBE) or a double-balloon enteroscopy (DBE) [4,5,6].

DBE was developed in 2001. Since then, it has become one of the standard methods in the diagnosis and treatment of small bowel (SB) disease [6,7,8]. This procedure not only allows observation of the SB mucosa, but allows performing biopsies, removing polyps, stopping bleeding, and dilating strictures [9,10]. Compared to spiral enteroscopy, DBE appears to allow for a deeper insertion [11]. In addition, although some studies have shown contradictory results, a prospective single-center study in Japan has shown that DBE has significantly better results than SBE in relation to complete enteroscopy [12]. 

Although DBE is an effective procedure for diagnosing and treating SB lesions, there might be a great difference in its usefulness depending on the experience and skill of the operator [1]. Although a number of studies have reported the diagnostic yield of DBE, most of them have been conducted using data from a multicenter, retrospective registry [13,14,15]. As a result, it is difficult to guarantee the reliability and accuracy of their results due to differences in skill level among operators and difficulties in data collection. Meanwhile, when a single-center study is conducted, it is difficult to secure a sufficient number of cases [7,16,17,18].

To compensate for these shortcomings, it is necessary to investigate the results of a single endoscopist with long experience. Thus, the aim of this study was to investigate the indication, location of the lesion, diagnostic yield, and therapeutic yield of DBE based on 17 years of experience of a single operator in a single center.

## 2. Materials and Methods

### 2.1. Patients

A single-center, retrospective study was performed. We reviewed medical records of subjects who received DBE from August 2004 to July 2021 at Hallym University Medical Center, Korea. Patients with inadequate endoscopic images or medical records and patients who were transferred to another hospital before final diagnosis were excluded from this study. In addition, cases with procedures performed by another endoscopist besides HJ Jang were excluded. 

Patients’ age, sex, underlying diseases, gastrointestinal surgery history, cancer history, and medication history were investigated. Underlying diseases included diabetes, hypertension, dyslipidemia, heart disease, cerebrovascular disease, chronic kidney failure, cirrhosis, obstructive pulmonary disease, asthma, and rheumatic disease. If the patient underwent surgery or had a biopsy, results were evaluated. Types and locations of lesions found during DBE, operation time, insertion route, complications, and final diagnosis were confirmed through chart review and endoscopy review. Even for the same patient, if the examination interval was more than 6 months, it was considered a new examination and included in this study.

This study was approved by the Institutional Review Board (IRB) of Hallym University School of Medicine (HDT 2021-04-011-001). It was performed in compliance with the ethical guidelines of the Declaration of Helsinki. All data were fully anonymized before we accessed them. This study was registered with the Clinical Research Information Service (CRIS) (ID: KCT0006267). 

### 2.2. Endoscopy

DBE (EN-450P5/20, EN-450T5, and EN-580T in chronological order; Fujinon Inc., Saitama, Japan) was performed by a single endoscopist (HJ Jang). All DBEs were performed under conscious sedation (midazolam with or without propofol and pethidine) administered by the endoscopist. Bowel preparation was not required for the oral route. Polyethylene glycol solution was used for the anal route. In patients with slow transit or a history of prior abdominal surgery, bowel preparation with a cleansing solution was sometimes used for oral DBE. Route of insertion was determined based on clinical features, capsule endoscopy (CE), or imaging study. If a lesion was observed in the prior diagnostic tests [CE, computed tomography (CT), etc.] and no lesion was found in the first insertion route, the test was performed again by another route. A ‘push and pull technique’ was used to pleat the SB onto the overtube. If the same patient underwent a DBE the next day by a different route, these two procedures were considered one procedure and the total procedure time was the sum of the two.

For an effective deep insertion of the scope without unwanted looping, the scope insertion was performed by a shortening procedure with fluoroscopy guidance. The insertion depth of the endoscope was estimated considering the number of shortenings and the location of the endoscopic tip on the fluoroscopy. In addition, if there was a lesion whose location was confirmed by other imaging tests, the insertion depth of the scope was determined by referring to the location. 

### 2.3. Definitions

Indication for BAE was defined as the primary reason for DBE. It was classified into obscure gastrointestinal bleeding (OGIB), unexplained chronic abdominal pain or diarrhea, abnormal findings on diagnostic imaging (including CE, CT, and/or magnetic resonance imaging), histological confirmation of a suspected disease, evaluation of the underlying disease, and foreign body removal. OGIB is defined as gastrointestinal bleeding from an unknown source that persists or recurs after repeated upper and lower endoscopy. In our OGIB cases, contrast-enhanced CT scan and/or CE was conducted to detect the source of bleeding and determine the insertion route prior to the DBE. If the focus of bleeding was not seen in the diagnostic evaluation and bleeding persisted, both anterograde and retrograde enteroscopy were planned to be performed. 

**Figure 1:**
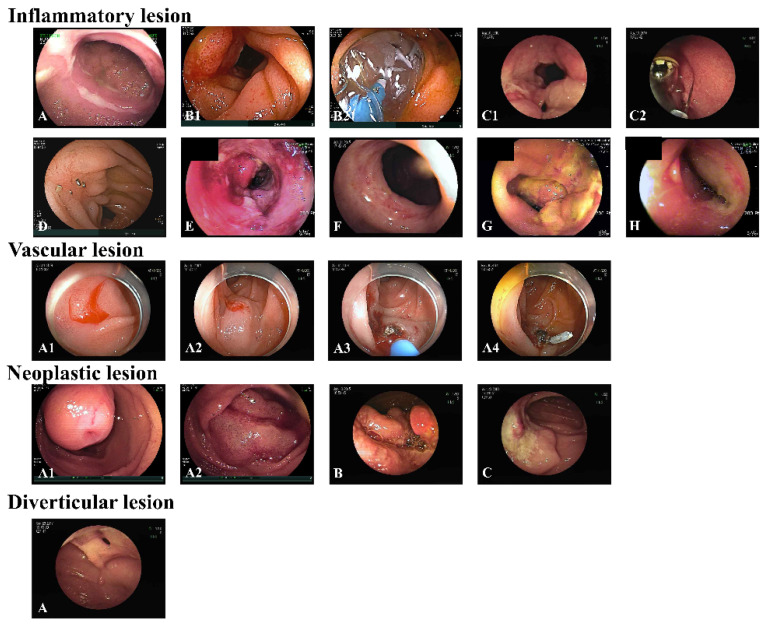
Endoscopic images of double-balloon enteroscopy. **Inflammatory lesions:** (**A**) Intestinal tuberculosis on mid-jejunum, (**B1**,**B2**) Crohn’s disease with luminal narrowing on mid-ileum, balloon dilatation, (**C1**,**C2**) Crohn’s disease with capsule retention, foreign body removal, (**D**) Postoperative stricture mid-jejunum, (**E**) Ischemic enteritis on distal ileum, (**F**) Cryptogenic multifocal ulcerous stenosing enteritis on proximal ileum, (**G**) Henoch–Schönlein purpura on proximal jejunum, (**H**) NSAID-induced enteropathy on distal ileum. **Vascular lesions:** (**A1**–**A4**) Dieulafoy’s lesion on duodenal 3rd portion, epinephrine injection, argon plasma coagulation, and hemoclipping. **Neoplastic lesions:** (**A1**,**A2**) Malignant gastrointestinal stromal tumor on proximal jejunum, tattooing before surgery, (**B**) Adenocarcinoma on proximal jejunum, (**C**) Burkitt’s lymphoma on distal jejunum. **Diverticular lesion:** (**A**) Meckel’s diverticulum on distal ileum.

Diagnostic yield was defined as the ratio of the number of patients with positive DBE findings. A positive DBE finding was defined as the presence of any significant positive endoscopic finding consistent with the patient’s clinical presentation. They were classified into inflammatory lesions (including erythema, erosions, and ulcers), vascular lesions (including angiodysplasia, Dieulafoy’s lesions, and arteriovenous malformations), neoplastic lesions (benign or malignant tumors and polyposis), diverticular lesions, and other lesions (foreign bodies) (Figure 1). A negative DBE finding was defined as a case where the lesion was not found through DBE or when scope insertion to the relevant site failed even though there was clear evidence of bleeding or tumor.

Locations of lesions were categorized into duodenum, proximal jejunum, mid-jejunum, distal jejunum, proximal ileum, mid-ileum, and distal ileum. In the case of multiple lesions, the location of the lesion was defined as the most proximal lesion in the anal approach and the most distal one in the oral approach or in both (anal and oral) approaches. 

When endoscopic treatment was performed during DBE, treatment methods were investigated. Treatment methods included polypectomy or endoscopic mucosal resection (EMR), argon plasma coagulation (APC), hemoclipping, epinephrine injection, tattooing before surgery, and foreign body removal. A positive therapeutic yield was defined as performance of any significant therapy excluding biopsies.

Complications were defined as serious adverse events that occurred during and after the procedure, including postoperative bleeding, bowel perforation, pancreatitis, and procedure-related death. Nausea, vomiting, abdominal distension, and other transient, self-limiting symptoms were not included as complications. Final diagnosis was defined as a diagnosis that was ultimately decided in consideration of other test results, clinical features, and surgical results.

### 2.4. Statistical Analysis

Continuous variables are expressed as median (interquartile range), whereas categorical variables are presented as frequency (%). Differences between positive and negative DBE finding groups were evaluated using the Mann–Whitney U-test for continuous data and chi-squared test or Fisher’s exact test for categorical data. A *p*-value of less than 0.05 was considered statistically significant. All statistical analyses were performed using SPSS version 19.0 for Windows (IBM Corp., Armonk, NY, USA).

## 3. Results

### 3.1. Patients and Indications

A total of 267 patients were included, of whom 98 (36.7%) were women. The overall mean age was 47.11 ± 16.95 (range 13–85) years. Numbers of those with a history of gastrointestinal surgery and a history of cancer were 26 (9.7%) and 10 (3.7%), respectively. Other characteristics of the study population are shown in Table 1. In 50 cases (21 patients), two or more DBE procedures were performed at least 6 months apart for the same patient. These 50 cases included 30 cases with Crohn’s disease (12 patients), 8 cases with Peutz–Jeghers syndrome (PJS, 3 patients), 4 cases with intestinal tuberculosis (2 patients), 6 cases with OGIB (3 patients), and 2 cases with malignant lymphoma (1 patient). 

Indications for DBE were OGIB in 123 (46.1%), unexplained chronic abdominal pain or diarrhea in 52 (19.5%), abnormal findings on diagnostic imaging in 36 (13.5%), histological confirmation of suspected disease in 12 (4.5%), evaluation of the underlying disease in 38 (14.2%), and foreign body removal in 6 (2.2%) patients (Table 2). 

### 3.2. Endoscopic Results

The procedure time ranged from 27 min to 250 min (mean: 58.39 ± 27.21 min, Table 2). Procedures were carried out using an anterograde approach in 96 cases, a retrograde approach in 114 cases, and both approaches in 57 cases. The types of lesions were inflammatory in 114 (42.7%), vascular in 19 (7.1%), neoplastic in 58 (21.7%), diverticular in 14 (5.2%), and other (foreign bodies) in 5 (1.9%). These lesions were found on the duodenum in 12 (4.5%), the proximal jejunum in 43 (16.1%), the mid-jejunum in 27 (10.1%), the distal jejunum in 18 (6.7%), the proximal ileum in 20 (7.5%), the mid-ileum in 17 (6.4%), and the distal ileum in 73 (27.3%) patients. Biopsies were performed for 113 (42.3%) cases. During and after the procedure, GI bleeding, bowel perforation, and pancreatitis occurred in 5, 1, and 1 case(s), respectively. No procedure-related deaths occurred. 

After the procedure, surgery for treatment or diagnosis was performed for 55 (20.6%) cases. The reasons for surgery were malignancy potential in 18, small bowel stricture or obstruction in 14, persistent GI bleeding in 21, foreign body removal in 1, and diagnostic evaluation in 1. The final diagnoses of the patients who underwent surgery are shown in Supplementary Table S1. In addition, there were 10 (3.7%) cases referred to other hospitals for surgery (5 malignant lymphoma, 2 adenocarcinoma, 2 Peutz–Jeghers syndrome, and 1 Meckel’s diverticulum).

### 3.3. Diagnostic and Therapeutic Yields

Overall diagnostic yield was 78.7% (210/267). Diagnostic yields for OGIB, unexplained chronic abdominal pain or diarrhea, abnormal findings on diagnostic imaging, histological confirmation of suspected disease, evaluation of underlying disease, and foreign body removal were 68.3% (84/123), 84.6% (44/52), 80.6% (29/36), 91.7% (11/12), 97.4% (37/38), and 83.3% (5/6), respectively (Supplementary Table S2). There were 7 cases where abnormal findings were suspected on imaging tests, but no lesions were found on DBE (Supplementary Table S3). Their final diagnoses were normal in 5, gastrointestinal stromal tumor in 1, and anastomosis site stricture in 1. The diagnostic yield for OGIB was significantly lower (*p* < 0.001) than for other indications, while the diagnostic yield for evaluating underlying diseases was significantly higher (*p* = 0.001, Table 3). The procedure time was longer in patients with negative DBE findings than in those with positive DBE findings (*p* = 0.013). However, there was no difference between the two groups in the number of patients with a procedure time greater than 90 min.

Therapeutic yield was 24.7% (66/267). Polypectomy (or EMR) was performed in 16 cases, APC in 11, hemoclipping in 20, epinephrine injection in 10, steroid injection in 1, balloon dilatation in 6, tattooing before surgery in 11, and foreign body removal in 6. In 13 cases, two or more therapies were performed during one procedure. Reasons for the treatment were hemostasis in 26 cases, tumor resection in 16, preoperative localization of lesion in 10, preoperative localization of lesion with hemostasis in 1, dilatation of narrowed lumen in 6, foreign body removal in 6, and alleviation of inflammation in 1. Results of polypectomy (or EMR) were hamartoma, hyperplastic polyp, adenoma, and brunneroma in 11, 3, 1, and 1 case(s), respectively. Failure of the treatment procedure occurred in two cases, both for foreign body removal.

### 3.4. The Location of Lesion and Final Diagnosis

Among inflammatory lesions, the most common final diagnosis was Crohn’s disease (56.1%), followed by non-specific erosion or ulcer (14.9%), intestinal tuberculosis (7.0%), nonsteroidal anti-inflammatory drug (NSAID) enteropathy (5.3%), and non-specific inflammation (5.3%, Table 4). For those with Crohn’s disease, intestinal tuberculosis, or NSAID enteropathy, lesions were mainly found in the ileum. Non-specific inflammation was mainly found in the jejunum and non-specific erosion or ulcer was found evenly throughout the small intestine. Vascular lesions were found predominantly in the jejunum. Among neoplastic lesions, the most common final diagnosis was PJS (22.4%), followed by malignant lymphoma, adenocarcinoma, and gastrointestinal stromal tumor (12.1% each). Neoplastic lesions were found more frequently in the jejunum than in the ileum. Diverticular lesions were more commonly found in the ileum, with 57.1% of diverticular lesions being Meckel’s diverticulum. The frequency of the diseases finally diagnosed is shown in Supplementary Table S1.

## 4. Discussion

In our study, the diagnostic yield of DBE was 78.7% and the most common indication for DBE was OGIB. DBE had various diagnostic rates according to indications of the test. These findings are not very different from those of previous studies [13,14,15,16,17,18]. According to a Korean multicenter retrospective DBE registry study published in 2007, indications for examination were OGIB in 61%, chronic abdominal pain in 14%, radiologic/capsule endoscopic abnormality in 11%, polyposis in 4%, and chronic diarrhea in 4%, with an overall diagnostic yield of 75% [15]. In 2016, a multicenter retrospective study using the BAE (DBE+SBE) registry was published again in Korea [14]. The overall diagnostic yield was similar (74.6%) and the most common indication was OGIB (58.3%). According to a meta-analysis, the overall diagnostic yield was 68.1% and the most common indication was suspected mid-GI bleeding (62.5%) [13].

DBE is a procedure with significant differences in technical proficiency among operators. For multicenter registry studies, operator heterogeneity may reduce the reliability of the results. However, since this study was about the procedures performed in a single center by a single operator, it was possible to sufficiently guarantee a high reliability of the collected data and the consistency of procedures performed.

Our study showed that the diagnostic yield for OGIB was significantly lower than for other indications. However, in many previous studies, the diagnostic yield for OGIB was not different from the overall diagnostic yield [13,14]. The lower diagnostic yield of OGIB in our study might be due to the following reasons: First, when a DBE is performed for OGIB, there might be cases where there is no actual lesion in the SB, or the lesion has already healed at the time of the examination. Thus, even if the DBE was performed well, a negative result might be shown. Second, as shown in our results, erosive or ulcerative lesions can occur anywhere in the SB, making them difficult to detect. Lastly, since bleeding sites are often difficult to locate clearly on imaging tests, it might be more difficult to detect a vascular lesion than other lesions (e.g., inflammatory lesion or neoplastic lesion). However, the diagnostic yield of OGIB in our study was not lower than that of other studies [13,14,15,16,17,18]. This was because the overall diagnostic yield in our study was relatively high. Additionally, in our study, the procedure time was longer in patients with negative DBE findings. The reason might be because if no lesion is found, longer endoscopic time is required to look for the lesion.

DBE is a relatively safe test with few complications. According to a previous meta-analysis, pooled minor and major complication rates were 9.1% and 0.72%, respectively [13]. Complication rate in our study was 2.6%. We did not include minor symptoms such as abdominal pain or vomiting as complications. Thus, the overall complication rate was low. In our experience, abdominal pain and vomiting are common complications after the procedure, but in most cases they resolve spontaneously. In our results, pancreatitis and bowel perforation occurred in one case each. However, there were no procedure-related deaths. 

In previous studies, the most widely used treatment was APC [16,19]. However, hemoclipping was most commonly performed in our study. This might be related to the tendency to prefer hemoclips for hemostasis in East Asia [20,21]. The most common reason for treatment was hemostasis, followed by polyp removal, marking the location of the lesion before surgery, widening a narrowed lumen, and removing foreign bodies. Since most treatments have been successful, DBE is considered to be an effective method for treatment as well as for diagnosis. However, it will be safer to try endoscopic treatment after sufficient experience of DBE has been accumulated.

Since the location where the lesion is often found is different for each disease, the insertion route for DBE should be determined differently depending on the suspected disease. In addition, depending on the location of the suspected lesion, different possible diagnoses can be inferred. In our study, looking at the correlation between the final diagnosis and the location of the lesion, Crohn’s disease, intestinal tuberculosis, NSAID enteropathy, and diverticular lesions were mainly found in the ileum, while vascular lesions, non-specific inflammation, and neoplastic lesions were found more frequently in the jejunum than in the ileum. On the other hand, non-specific erosions or ulcers were found evenly throughout the SB. 

This study has some limitations. First, the sample size of our study might not be large when compared to a multicenter study. To compensate for this shortcoming, we collected data over a long period of time. Second, indications for DBE are very diverse and heterogeneous. Methods for classifying indications can also vary [22,23,24,25], making it difficult to interpret the results. Therefore, we tried to classify reasons for the test as rationally as possible. However, there were cases where two or more indications coexisted. In such cases, the indication that occurred first was selected. In addition, the classification according to the nature of the lesion was also very vague in some cases. Even when bleeding focus was confirmed, if an inflammatory lesion was dominant, it was classified as an inflammatory lesion rather than a vascular lesion. Third, as this study is a retrospective study, it was impossible to confirm all detailed information about the patient and procedure. The incidence of complications might have been underestimated as minor symptoms were not included as complications in our study. This is because chart review alone could not detect all minor symptoms or signs. Lastly, the detection rate of lesions was independent of the actual prevalence. Among neoplastic lesions, the most common final diagnosis was PJS (22.4%). However, this result was independent of the incidence of the disease, since each patient with PJS was tested several times over the years.

In conclusion, the overall diagnostic yield of DBE was relatively high, and the diagnostic yield varied according to indications. The most common indication for DBE was OGIB. DBE had a lower diagnostic yield for OGIB than for other indications. Depending on the type of lesion, the location where it was commonly found was different. Most endoscopic treatments during DBE were successful and severe complications were rare. Thus, DBE is an excellent and safe endoscopic method for the diagnosis and treatment of SB lesions. 

## Figures and Tables

**Table 1:** Baseline characteristics of patients.

Variables	n = 267
**Age,** years *	46 (33–59)
**Female sex,** n (%)	98 (36.7)
**History of gastrointestinal surgery** ^†^, n (%)	26 (9.7)
**Comorbidity**, n (%)	
History of cancer	10 (3.7)
GI cancer/Other cancer	6/4
Diabetes	28 (10.5)
Hypertension	61 (22.8)
Dyslipidemia	42 (15.7)
Chronic kidney disease	10 (3.7)
Liver cirrhosis	13 (4.9)
Chronic obstructive pulmonary disease/Asthma	1 (0.4)/0
Rheumatoid arthritis/Ankylosing spondylitis	1 (0.4)/0
Heart disease	14 (5.2)
Stroke	7 (2.6)
**Medication**, n (%)	
Antiplatelet agent	21 (7.9)
Anticoagulant	9 (3.4)
NSAIDs	20 (7.5)
Oral steroid	3 (1.1)

* Continuous variables are presented as median (interquartile range). All other data are presented as number (%). ^†^ Appendectomies, hemorrhoid operations, hernia operations, and laparoscopic cholecystectomies were excluded from the history of GI surgery. GI, gastrointestinal; NSAIDs, nonsteroidal anti-inflammatory drugs.

**Table 2:** Indications and results of double-balloon enteroscopy.

Variables	n = 267
**Indication for the test,** n (%)	
Obscure GI bleeding	123 (46.1)
Unexplained chronic abdominal pain or diarrhea	52 (19.5)
Abnormal findings on diagnostic imaging	36 (13.5)
Histological confirmation of suspected disease	12 (4.5)
Evaluation of underlying disease	38 (14.2)
Foreign body removal	6 (2.2)
**Examination time,** min *	55 (38–69)
**Insertion route,** n (%)	
Anterograde approach	96 (36.0)
Retrograde approach	114 (42.7)
Both mouth and anus	57 (21.3)
**Diagnostic yield,** n (%)	210 (78.7)
**Frequency of diagnostic findings,** n (%)	
Inflammatory lesion	114 (42.7)
Vascular lesion	19 (7.1)
Neoplastic lesion	58 (21.7)
Diverticular lesion	14 (5.2)
Foreign body	5 (1.9)
Negative finding	57 (21.3)
**Biopsy**	113 (42.3)
**Location of the lesion**	
Duodenum	12 (4.5)
Proximal jejunum	43 (16.1)
Mid-jejunum	27 (10.1)
Distal jejunum	18 (6.7)
Proximal ileum	20 (7.5)
Mid-ileum	17 (6.4)
Distal ileum	73 (27.3)
No lesion	57 (21.3)
**Therapeutic yield,** n (%)	66 (24.7)
**Frequency of performed therapy,** n (%)	
Polypectomy or endoscopic mucosal resection	16 (6.0)
Argon plasma coagulation	11 (4.1)
Hemoclipping	20 (7.5)
Epinephrine injection	10 (3.7)
Steroid injection	1 (0.4)
Balloon dilatation	6 (2.2)
Tattooing before surgery	11 (4.1)
Foreign body removal	6 (2.2)
**Procedure-related complications,** n (%)	7 (2.6)
Bleeding/Bowel perforation/Pancreatitis	5/1/1
Procedure-related death	0
**Surgery after enteroscopy,** n (%)	55 (20.6)
**Reason for the surgery**	
Malignancy potential	18
Small bowel stricture or obstruction	14
Persistent GI bleeding	21
Foreign body removal	1
Diagnostic evaluation	1
**Referred to another hospital for surgery**	10 (3.7)

* Continuous variables are presented as median (interquartile range). All other data are presented as number (%).

**Table 3:** Comparison between patients with positive DBE finding and those with negative DBE finding.

Variables	Positive DBE Finding (n = 210)	Negative DBE Finding (n = 57)	*p*-Value
Age, years *	45 (33–59)	50 (36–61)	0.231
≥60 years	48 (22.9)	18 (31.6)	0.225
**Female sex**	72 (34.3)	26 (45.6)	0.124
**Comorbidity**			
History of cancer	7 (3.4)	3 (5.3)	0.379
Diabetes	21 (10.0)	7 (12.3)	0.628
Hypertension	49 (23.3)	12 (21.1)	0.859
Dyslipidemia	35 (16.7)	7 (12.3)	0.539
Chronic kidney disease	10 (4.8)	0	0.126
Liver cirrhosis	8 (3.8)	5 (8.8)	0.159
Heart disease	13 (6.2)	1 (1.8)	0.314
**Medication**			
Antiplatelet agent	15 (7.1)	6 (10.5)	0.409
Anticoagulant	5 (2.4)	4 (7.0)	0.101
NSAIDs	16 (7.6)	4 (7.0)	1.000
**Indication**			
Obscure GI bleeding	84 (40.0)	39 (68.4)	<0.001
Unexplained chronic abdominal pain or diarrhea	44 (21.0)	8 (14.0)	0.345
Abnormal findings on diagnostic imaging	29 (13.8)	7 (12.3)	1.000
Histological confirmation of suspected disease	11 (5.2)	1 (1.8)	0.471
Evaluation of underlying disease	37 (17.6)	1 (1.8)	0.001
Foreign body removal	5 (2.4)	1 (1.8)	1.000
**History of gastrointestinal surgery** ^†^	21 (10.0)	5 (8.8)	1.000
Procedure time, min *	52 (36–68)	60 (48.5–75.5)	0.013
≥90 min	22 (10.5)	7 (12.3)	0.640

* Continuous variables are presented as median (interquartile range) and analyzed by Mann–Whitney test. All other data are presented as number (%) and analyzed by chi-squared test or Fisher’s exact test. ^†^ Appendectomies, hemorrhoid operations, hernia operations, and laparoscopic cholecystectomies were excluded from the history of gastrointestinal surgery. DBE, double-balloon enteroscopy; NSAIDs, nonsteroidal anti-inflammatory drugs.

**Table 4:** Relationship between final diagnosis and location of the lesion.

Endoscopic Findings and Final Diagnosis	Location of the Lesion							
	Duodenum, n (%)	Proximal Jejunum, n (%)	Mid-Jejunum, n (%)	Distal Jejunum, n (%)	Proximal Ileum, n (%)	Mid-Ileum, n (%)	Distal Ileum, n (%)	Total
**Inflammatory lesion**	2 (1.8)	8 (7.0)	10 (8.8)	8 (7.0)	14 (12.3)	12 (10.5)	60 (52.6)	114
Crohn’s disease	1 (1.6)	0	3 (4.8)	4 (6.5)	6 (9.7)	6 (9.7)	42 (67.7)	62
Intestinal tuberculosis	0	0	1 (12.5)	1 (12.5)	2 (25)	1 (12.5)	3 (37.5)	8
NSAID enteropathy	0	0	0	0	2 (33.3)	2 (33.3)	2 (33.3)	6
Behcet’s disease	0	0	0	0	0	0	1 (100)	1
HS purpura	0	1 (100)	0	0	0	0	0	1
Eosinophilic enteritis	0	0	0	1 (50)	0	0	1 (50)	2
Ischemic enteritis	0	0	0	0	1 (33.3)	0	2 (66.7)	3
CMUSE	0	0	0	0	0	0	1 (100)	1
Non-specific erosion or ulcer	1 (5.9)	4 (23.5)	3 (17.6)	1 (5.9)	3 (17.6)	1 (5.9)	4 (23.5)	17
Stricture of unknown cause	0	0	0	0	0	0	2 (100)	2
Non-specific inflammation	0	3 (50)	1 (16.7)	1 (16.7)	0	0	1 (16.7)	6
Anastomosis site ulcer or stricture	0	0	2 (40)	0	0	2 (40)	1 (20)	5
**Vascular lesion**	3 (15.8)	5 (26.3)	5 (26.3)	3 (15.8)	1 (5.3)	1 (5.3)	1 (5.3)	19
Angiodysplasia, AV malformation, or Dieulafoy’s lesion	3 (17.6)	4 (23.5)	4 (23.5)	3 (17.6)	1 (5.9)	1 (5.9)	1 (5.9)	17
Hemangioma	0	1 (50)	1 (50)	0	0	0	0	2
**Neoplastic lesion**	5 (8.6)	26 (44.8)	12 (20.7)	5 (8.6)	2 (3.4)	2 (3.4)	6 (10.3)	58
Malignant lymphoma	0	1 (14.3)	2 (28.6)	2 (28.6)	1 (14.3)	0	1 (14.3)	7
Adenocarcinoma	0	6 (85.7)	0	0	0	0	1 (14.3)	7
Adenomatous polyp	0	1 (100)	0	0	0	0	0	1
Hyperplastic polyp	0	1 (33.3)	1 (33.3)	0	0	0	1 (33.3)	3
Hamartomatous polyp (Except PJS)	0	1 (50)	0	0	0	0	1 (50)	2
PJS	1 (7.7)	8 (61.5)	3 (23.1)	0	0	1 (7.7)	0	13
GIST	2 (28.6)	2 (28.6)	1 (14.3)	1 (14.3)	0	0	1 (14.3)	7
Leiomyoma	0	0	0	2 (100)	0	0	0	2
Lipoma	0	2 (40)	1 (20)	0	1 (20)	1 (20)	0	5
Ectopic pancreas	1 (25)	1 (25)	1 (25)	0	0	0	1 (25)	4
Adenomyoma	0	1 (100)	0	0	0	0	0	1
Brunneroma	1 (100)	0	0	0	0	0	0	1
Lymphangioma or lymphangiectasia	0	1 (50)	1 (50)	0	0	0	0	2
Subepithelial lesion without histologic confirmation	0	1 (33.3)	2 (66.7)	0	0	0	0	3
**Diverticular lesion**	1 (7.1)	3 (21.4)	0	0	2 (14.3)	2 (14.3)	6 (42.9)	14
Meckel’s diverticulum	0	0	0	0	1 (12.5)	2 (25)	5 (62.5)	8
Other diverticulum	1 (16.7)	3 (50)	0	0	1 (16.7)	0	1 (16.7)	6
**Foreign body**	1 (20)	1 (20)	0	2 (40)	1 (20)	0	0	5

NSAID, nonsteroidal anti-inflammatory drug; HS purpura, Henoch–Schönlein purpura; CMUSE, cryptogenic multifocal ulcerous stenosing enteritis; AV malformation, arteriovenous malformation; PJS, Peutz–Jeghers syndrome; GIST, gastrointestinal stromal tumor.

## Supplementary Materials

The following supporting information can be downloaded at: https://www.mdpi.com/article/10.3390/diagnostics12092224/s1, Table S1: Final diagnosis and surgical treatment; Table S2: Types of endoscopic findings and diagnostic yield according to individual indications; Table S3: Final diagnosis for patients with negative DBE finding according to the indications.

## Abbreviations

DBE, double-balloon enteroscopy; SBE, single-balloon enteroscopy; BAE, balloon-assisted enteroscopy; SB, small bowel; CE, capsule endoscopy; EMR, endoscopic mucosal resection; APC, argon plasma coagulation; PJS, Peutz–Jeghers syndrome; NSAID, nonsteroidal anti-inflammatory drug; OGIB, obscure gastrointestinal bleeding.
