# Supplementary material for: Indication, Location of the Lesion, Diagnostic Yield, and Therapeutic Yield of Double-Balloon Enteroscopy: Seventeen Years of Experience
Source: Diagnostics (Basel). 2022 Sep 14;12(9):2224. doi: 10.3390/diagnostics12092224 (PMC9498053; doi:10.3390/diagnostics12092224)
Supplement: Supplementary file 1 [file diagnostics-12-02224-s001.zip › diagnostics-1864812-supplementary.pdf]

Supplementary Table S1. Final diagnosis and surgical treatment.

Final diagnosis†	n (%) Total n=267	Surgery after enteroscopy, n (%) Total n=55	Referred to another hospital for surgery, n (%) Total n=10
Crohn's disease	63 (23.6)	5 (9.1)	
Angiodysplasia, AV malformation, or Dieulafoy's lesion	17 (6.4)	5 (9.1)	
Intestinal tuberculosis	8 (3.0)	2 (3.6)	
Behcet's disease	1 (0.4)	1 (1.8)	
HS purpura	1 (0.4)		
Eosinophilic enteritis	2 (0.8)	1 (1.8)	
Ischemic enteritis	3 (1.2)		
CMUSE	1 (0.4)		
Peutz-Jeghers syndrome	13 (4.9)		2 (20)
Hamartomatous polyp (Except Peutz-Jeghers syndrome)	2 (0.7)	1 (1.8)	
Meckel's diverticulum	9 (3.4)	8 (14.5)	1 (10)
Other diverticulum	6 (2.2)	1 (1.8)	
Foreign body	6 (2.2)	1 (1.8)	
GIST	8 (3.0)	8 (14.5)	
Leiomyoma	2 (0.7)	2 (3.6)	
Ectopic pancreas	4 (1.5)	3 (5.5)	
Lipoma	5 (1.9)	3 (5.5)	
Adenocarcinoma	7 (2.7)	5 (9.1)	2 (20)
Adenomatous polyp	1 (0.4)		
Malignant lymphoma	7 (2.7)	2 (3.6)	5 (50)
Hyperplastic polyp	3 (1.2)		
Adenomyoma	1 (0.4)	1 (1.8)	

Brunneroma	1 (0.4)	
Lymphangioma or lymphangiectasia	2 (0.7)	
Hemangioma	2 (0.7)	1 (1.8)
Anastomosis site stricture	7 (2.6)	1 (1.8)
Anastomosis site ulcer	1 (0.4)	
NSAID enteropathy	6 (2.2)	
Non-specific erosion or ulcer	17 (6.4)	2 (3.6)
Stricture of unknown cause	2 (0.7)	1 (1.8)
Non-specific inflammation	6 (2.2)	1 (1.8)
Subepithelial lesion without histologic confirmation	3 (1.1)	
No abnormal finding or no bleeding focus	49 (18.4)	

† The final diagnosis refers to a diagnosis that is finally decided in consideration of other test results, clinical features, and surgical results.

AV malformation, arteriovenous malformation; HS purpura, Henoch–Schönlein purpura; CMUSE, Cryptogenic Multifocal Ulcerous Stenosing Enteritis; GIST, Gastrointestinal stromal tumor; NSAID, nonsteroidal anti-inflammatory drug.

Supplementary Table S2. Types of endoscopic findings and diagnostic yield according to individual indications.

Types of endoscopic findings	Indications					
	Obscure GI bleeding, n (%)	Unexplained chronic abdominal pain or diarrhea, n (%)	Abnormal findings on diagnostic imaging, n (%)	Histological confirmation of suspected disease, n (%)	To evaluate the underlying disease, n (%)	Foreign body removal, n (%)
**Inflammatory lesion**	37 (30.1)	33 (63.5)	12 (33.3)	10 (83.3)	22 (57.9)	0
**Vascular lesion**	17 (13.8)	1 (1.9)	1 (2.8)	0	0	0
**Neoplastic lesion**	19 (15.4)	9 (17.3)	14 (38.9)	1 (8.3)	15 (39.5)	0
**Diverticular lesion**	11 (8.9)	1 (1.9)	2 (5.6)	0	0	0
**Foreign body**	0	0	0	0	0	5 (83.3)
**Negative finding**	39 (31.7)	8 (15.4)	7 (19.4)	1 (8.3)	1 (2.6)	1 (16.7)
**Total**	123	52	36	12	38	6
**Diagnostic yield**	68.3%	84.6%	80.6%	91.7%	97.4%	83.3%

Supplementary Table S3. Final diagnosis for patients with negative DBE finding according to the indications.

Final diagnosis for patients with negative DBE finding	Indications of patients with negative DBE finding					
	**Obscure GI bleeding (Total n=39)**	**Unexplained chronic abdominal pain or diarrhea (Total n=8)**	**Abnormal findings on diagnostic imaging (Total n=7)**	**Histological confirmation of suspected disease (Total n=1)**	**To evaluate the underlying disease (Total n=1)**	**Foreign body removal (Total n=1)**
No abnormal finding or no bleeding focus	36	7	5	0	1	0
Meckel's diverticulum	1	0	0	0	0	0
Other diverticulum	1	0	0	0	0	0
Anastomosis site stricture	1	1	1	0	0	0
GIST	0	0	1	0	0	0
Crohn's disease	0	0	0	1	0	0
Foreign body	0	0	0	0	0	1

GIST, Gastrointestinal stromal tumor
